# Climate Change and Pediatric Gastrointestinal Health: A Narrative Review

**DOI:** 10.1016/j.gastha.2026.101116

**Published:** 2026-09-17

**Authors:** Amanda Kuhn, Sonal Dugar, Meera Shah, Rebecca Philipsborn

**Affiliations:** 1 Emory University Department of Pediatrics, Atlanta, Georgia; 2 Children’s Healthcare of Atlanta, Atlanta, Georgia; 3 Children’s Healthcare of Atlanta, Division of Gastroenterology, Atlanta, Georgia; 4 Advocate Health, Levine Children’s Hospital, Department of Pediatric Gastroenterology, Charlotte, North Carolina; 5 Gangarosa Department of Environmental Health, Emory University Rollins School of Public Health, Atlanta, Georgia

**Keywords:** Environmental Health, Pediatrics, Climate Change, Gastrointestinal Health, Sustainability

## Abstract

In 2025, the United Nations acknowledged that the world has failed to reduce carbon emissions sufficiently to avoid 1.5C warming—the level set by the Paris Accords to avoid devastating consequences. Communities already feel the implications of climate change, and it is creating new challenges for patients and health-care teams. While global efforts to reduce emissions and prevent the worst consequences of climate change persist, efforts to understand and adapt to the current and near-term consequences of climate change are also vital. Climate change is contributing to more frequent and severe weather events and compounding other ecological changes, with immediate and downstream implications for health. Pediatric patients are particularly vulnerable to the impacts of climate change, given their physiology, behaviors, ongoing development, and dependence on caregivers. This narrative review focuses on the implications of climate change for pediatric gastrointestinal (GI) health, elucidating key pathways by which climate change intersects with GI health that emerge from the literature. These pathways include heat-related illness and prenatal exposure to extreme heat, health implications of climate-driven migration and displacement, changing patterns of infectious GI diseases, the effects of pollution on noncommunicable GI diseases, the impacts of climate change on mental health and the gut-brain axis, and exacerbation of food insecurity and malnutrition. By understanding risks to their patients from these pathways, pediatric GI professionals may be better poised to assess for climate-driven exposures and to address patient care needs related to climate change. Pediatric GI professionals also have an opportunity as a community to engage in the needed health-care sector transformation to sustainable care delivery, bolster the resilience of health-care infrastructure, and advocate for policies to protect pediatric patients.

## Introduction

Climate change is presenting new and evolving challenges for pediatric gastrointestinal (GI) health.^1^ The serious and multifaceted implications of climate change for child health span age ranges, organ systems, and disease processes.^2^^,^^3^ The UNICEF Children’s Climate Risk Index names climate change as one of the direst threats facing children globally with an estimated 1 billion children at extremely high risk to these impacts.^4^ Compared to children born in 1960, those born in 2020 are 2.8 times as likely to be exposed to crop failures, 2.6 times as likely to be exposed to droughts, 2.8 times as likely to be exposed to river floods, and 6.8 times as likely to be exposed to heatwaves in their lifetime.^5^ Children most at risk live in the countries that are least responsible for the climate crisis: the 33 countries at highest risk emit only 9 percent of global carbon dioxide (CO_2_) emissions.^4^ The direct impacts of climate change—ranging from extreme heat to severe weather—are levers on the incidence and severity of common pathologies and the global burden of childhood illnesses.^6^^,^^7^

The urgency and enormity of the climate crisis may seem distant from the health-care needs of individual pediatric patients and the daily role of clinicians caring for children with GI disease. In reality, climate change influences an individual child’s risk of exposure to environmental hazards and ability to access health care. Some of these risks of climate change for GI health have been enumerated for gastroenterologists caring for adult patients.^8^

In this narrative review, we describe the major pathways through which climate change can affect pediatric GI health (Table, Figure 1). These pathways include: heat-related illness and prenatal exposure to extreme heat, effects of pollution on noncommunicable GI diseases, health implications of climate-driven migration and displacement, changing patterns of infectious GI diseases, impacts on mental health and the gut-brain axis, and exacerbation of food insecurity and malnutrition. By understanding risks to their patients from these pathways, pediatric GI professionals may be better poised to prepare for and assess climate-driven exposures, as well as to address patient-care needs related to climate change. Outside of the clinical setting, GI health-care professionals can lend unique expertise to systems-based solutions that promote the well-being of children in the climate crisis.

**Table:** Climate Change Effects and Associations With Pediatric Gastrointestinal Health

Climate change effects	GI health associations
Extreme heat	Hepatic injury, preterm birth (potentially higher risk of NEC given premature birth), increased risk of IBD flares
Environmental pollution (eg, ozone, particulate matter, agricultural runoff)	Preterm birth, higher incidence of IBD, MASLD, and FGIDs, harmful algae blooms (can contribute to dysbiosis, hepatotoxicity)
Increased precipitation and severe weather events	Increase in diarrheal (eg, *Campylobacter*), waterborne (eg, *Vibrio*) and vector-borne diseases (eg, malaria, dengue), reduced breastfeeding/increased formula feeding, forced migration, disruptions in health care (eg, accessing care or specialized equipment), dysbiosis
Strain on agriculture, changes in food availability, biodiversity loss	Reduced breastfeeding/increased formula feeding, dysbiosis, childhood obesity
Climate anxiety	Increase incidence of FGIDs, substance use with GI implications (eg, alcohol-related GI disease), increased risk of IBD flares, childhood obesity

**Figure 1:**
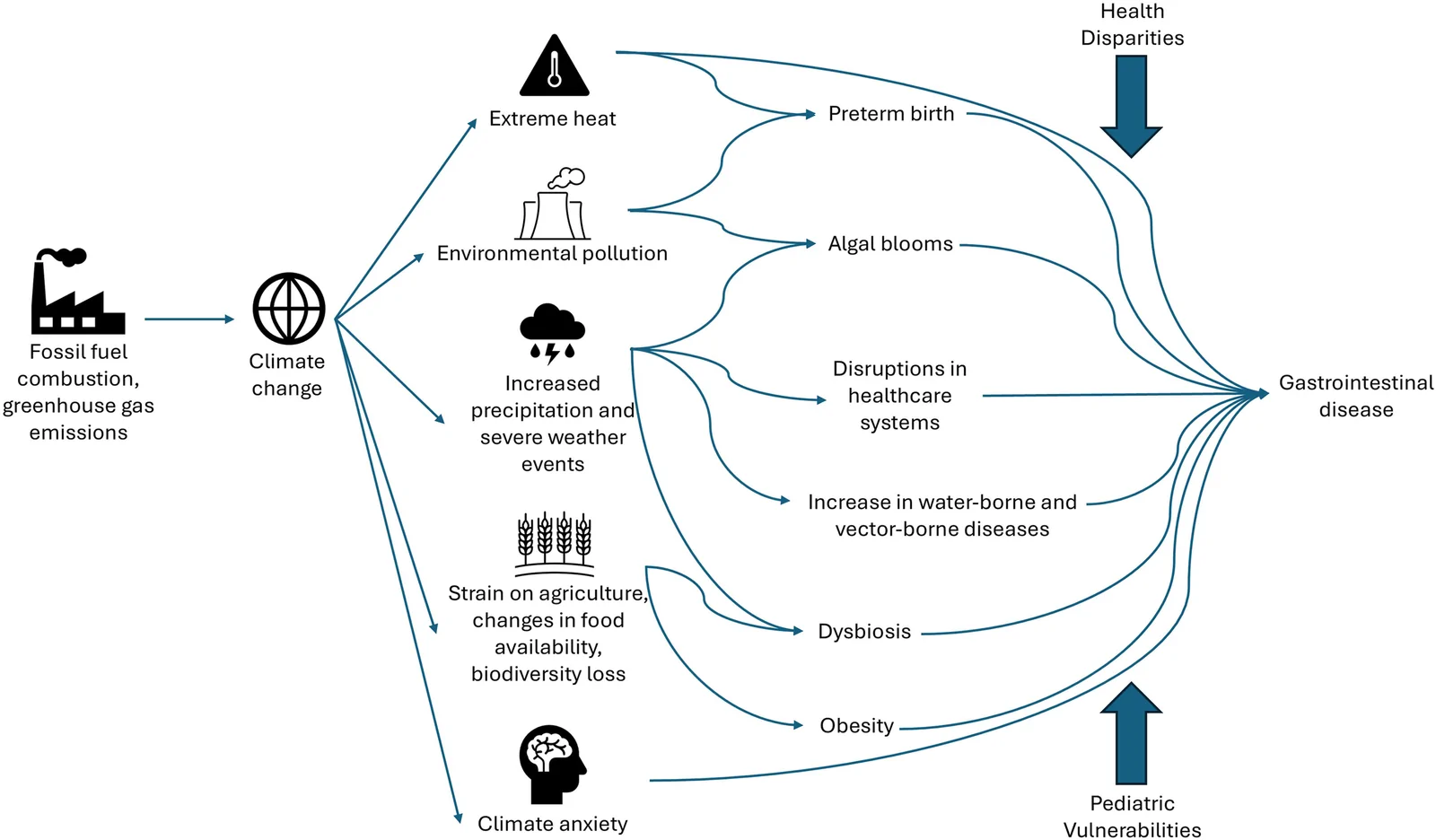
Mechanisms of climate change effects and associations with pediatric gastrointestinal disease.

### Heat-Related Illness and Perinatal Implications of Extreme Heat

More days of extreme heat due to climate change pose a direct threat to child health. Infants and young children are particularly at risk of heat-related illness due to their reduced ability to control their surroundings (eg, clothing, water intake, immediate environment), physiological differences in thermoregulation, and longer time required to acclimate to extreme heat.^9^ Extreme heat also increases a child’s risk of dehydration. For children using medications that affect fluid homeostasis, such as certain laxatives commonly prescribed by GI professionals, this risk may be increased.

Acute exposure to extreme heat can lead to hepatic, pancreas, and intestinal injury. While pediatric research on the topic is scarce, studies focusing on adults support acute liver injury secondary to heat-related illness.^10^^,^^11^ Proposed mechanisms include ischemia secondary to splanchnic vasoconstriction and direct cytotoxic effects of heat.^8^^,^^12^, ^13^, ^14^ Vasoconstriction and direct cytotoxic mechanisms can also contribute to heat stress–related intestinal damage, leading to increased permeability of the intestinal epithelium, resultant bacterial and toxin translocation, and systemic inflammatory response.^12^, ^13^, ^14^ Individuals with autoimmune GI illness are among those susceptible to flares through these pathways. As an example, a retrospective study in Zurich from 2001 to 2005, including older adolescents and adults, identified the presence of a heat wave as increasing the risk of hospitalization for inflammatory bowel disease (IBD) flares by 4.6%.^15^

Extreme heat also affects the health of the developing gut—even before birth. Babies born prematurely have greater risk of mortality and morbidity, including GI concerns, in the perinatal period compared to those born full-term. In a systematic review of studies including over 32 million US births, there was a significant association between maternal exposures to extreme heat and air pollutants, including ozone (O3) or fine particulate matter, and preterm birth, low birth weight, and stillbirth, with Black mothers at increased risk.^16^ A literature review evaluating racial disparities in climate-related health effects in the United States echoed that climate change is exacerbating socioeconomic drivers of existing health disparities for children and women of color.^17^ These implications of climate change on perinatal outcomes illustrate how compounding exposures can perpetuate health inequities across generations. Proposed mechanisms by which maternal heat exposure contributes to these adverse outcomes is through heat stress, premature labor, and a heat-associated increase in risk of maternal hypertensive disorders.^18^ Exposure to heat causes physiological stress via several hypothesized mechanisms that can affect both the fetus and placenta, including oxidative stress, changes in metabolism, cell-level derangements to mitochondria and endothelium, and inflammation.^19^

Necrotizing enterocolitis (NEC) is a GI illness occurring most commonly in preterm infants and with high morbidity and mortality. The etiology of NEC is multifactorial, including contributions from gut immaturity, vascular tone, and an abnormal intestinal microbiome.^20^ While there have been no studies to date that directly measure the effects of maternal heat exposure during pregnancy or infant heat exposure in the early newborn period on subsequent risk of developing NEC, the discussed associations between climate-mediated factors and risk of prematurity highlights climate change as an important and understudied population-level risk factor.

Climate-driven extreme weather may also indirectly modulate other NEC risk factors, such as infant feeding. The benefits of breast-feeding for infant gut microbiota, immune development, and overall health are well-documented, whereas formula feeding is associated with increased risk of NEC.^21^ A prospective cohort study of women in Burkina Faso finds that higher ambient temperatures may decrease a mother’s time spent breastfeeding and her likelihood of exclusively breastfeeding a young infant.^22^ Burkina Faso is classified by the World Bank as a low-income country and has average climates ranging from 25 to 33 °C, so these findings may not be generalizable to other settings. Nevertheless, they reflect a potential point of intervention: supporting breastfeeding in hot climates. Climate-driven disasters may also impinge upon breastfeeding success. In a study evaluating the effects of large-scale evacuation on infant feeding after the 2016 Fort McMurray wildfire, there was an overall reduction in breastfeeding rates and increase in use of infant formula, with some mothers reporting lack of lactation supports or safe places to feed or pump,^23^ findings that have been echoed in a recent review investigating feeding challenges during natural disasters.^24^

Other systemic manifestations of prenatal heat exposure include an association with cardiac congenital anomalies.^25^ There is sparse and conflicting evidence regarding maternal hyperthermia or ambient extreme heat and congenital GI anomalies such as abdominal wall defects at birth.^26^

### Impacts of Environmental Pollution on Autoimmune and Noncommunicable Diseases

Many noncommunicable diseases, including GI diseases, are influenced by both genetic factors and environmental exposures. Nearly the entire world’s human population (99%) breathes air with pollution that exceeds WHO recommended levels.^27^ In turn, local air quality is typically worse on hot days—which are increasing due to climate change. Fires fueled by climate change, such as the Los Angeles fires in 2025, are also worsening air quality.^28^^,^^29^ Pollutants alter immunity through various mechanisms, including epigenetic effects on immune cells, influences on gut and lung microbiome, inflammation of epithelial and mucosal barriers, and direct toxicity.^30^ The respiratory effects of air pollution are well-described, with pollution contributing to inflammation of the respiratory tract, as well as exposure-associated development and exacerbation of conditions such as asthma and allergic rhinosinusitis.^31^ Although less well-characterized, air pollution also contributes to inflammation in the GI tract, with studies observing increased permeability through widened junction spaces in the epithelial barrier, increased interleukin‑6 expression, and apoptosis of intestinal epithelial cells.^32^

A defective epithelial barrier or “leaky gut” can compromise the body’s immune defense and increases the risk for diagnosis and exacerbation of autoimmune conditions such as IBD, celiac disease, or eosinophilic esophagitis (EoE).^33^ EoE is hypothesized to be a result of genetic predisposition in conjunction with exposure to environmental factors. Multiple factors including maternal fever, preterm labor, neonatal exposures such as cold climate, indoor pollutants, and breastfeeding vs formula use may all play a role in increased risk of development of EoE.^34^ The increased incidence of atopic diseases in patients who have EoE has led researchers to hypothesize about shared environmental risk factors for these diseases, particularly early life exposures.^35^ While emerging literature points to plausible associations between EoE and air pollution, aeroallergens, and climate-driven exposures, significant gaps remain in understanding these linkages.^35^

The current theory encompassing the pathogenesis of IBD includes a genetic predisposition acting in conjunction with a variety of environmental risk factors, including air pollution, water pollution, gut microbiome changes, exposures to certain microorganisms, diet, and geographical risk factors.^36^ These environmental risk factors can increase not only the risk of IBD but also the risk of disease flares. In a UK case control study, individuals ≤23 years of age who lived in areas with higher concentrations of nitrogen dioxide (NO2) in their living environment were more than twice as likely to be diagnosed with Crohn’s disease compared to those with lower NO_2_ exposure after accounting for confounding variables; this association was not observed in middle-aged individuals, suggesting possible age-specific vulnerabilities in youth.^37^ A separate pediatric retrospective cohort study in Canada demonstrated an association between exposure to oxidant capacity (calculated oxidative potential of NO_2_ and O3) and pediatric-onset IBD.^38^ Endocrine-disrupting chemicals can impact proliferator-activated receptor gamma and glucocorticoid receptors which have been suggested to play an important role in the pathogenesis of IBD.^39^ From a gut microbiome standpoint, bifidobacteria and firmicutes levels are low in patients with new onset IBD and flares.^39^ Further research is needed to better elucidate the relative contributions of and interplay between specific environmental factors in IBD onset and outcomes.

### Air Pollution and Metabolic Dysfunction–Associated Steatotic Liver Disease

Ambient air pollution has also been associated with childhood obesity and risk for metabolic dysfunction–associated steatotic liver disease (MASLD). MASLD has an estimated prevalence among children globally of 5%–10%,^40^ with a prevalence of 41.2% among children and adolescents with overweight and/or obesity.^41^ A cross-sectional analysis conducted in China demonstrated a correlation between pollutant exposure (NO_2_, sulfur dioxide, and fine particulate matter [2.5 microns or less in diameter]) and increased obesity rates in school-aged children, thought to be due to oxidative stress and adipose tissue inflammation.^42^ Another proposed mechanism is via pollution-mediated changes in the gut microbiome and fecal metabolome (metabolites in feces) in early development.^43^ In a cohort of Latino children, Alderete et al^43^ noted associations between prenatal and early-life air pollution exposure and gut microbiome and fecal metabolite changes, many of which have been associated with childhood obesity. More specifically to MASLD, in a group of young adults from the Southern California Children’s Health Study, a positive association was found between prior-month exposure to redox-weighted oxidative capacity (an exposure variable derived from ambient air pollutant levels of NO_2_ and O3 measuring their potential to cause oxidative injury) and risk of MASLD after adjusting for factors such as age, sex, ethnicity, and body mass index.^44^

Studies suggest that environmental factors are contributing not only to increased risk of MASLD but also the disease progression to steatohepatitis.^45^ In a study of Latino youth with obesity in California, exposure to certain air pollutants was positively associated with hepatic fat fraction and liver stiffness as measured by magnetic resonance imaging.^46^ A hypothesized molecular mechanism for this association is that certain pollutants (eg, particulate matter pollution and polychlorinated compounds) contribute to hepatic steatosis and impairment of liver metabolic pathways by causing Kupffer cell activation, which promotes inflammatory signaling pathways and contributes to oxidative stress.^47^ As exposure to air pollution may increase risk of the development and exacerbation of noncommunicable GI diseases, pediatric GI providers serve an important role in considering these exposures in the patient populations they care for.

### Rise of Infectious Gastrointestinal Diseases in a Changing Climate

#### Diarrheal diseases

Climate change also influences the spread of diarrheal diseases through multiple mechanisms. Climate-driven extreme weather events affect water, sanitation, and hygiene infrastructure, and rising global temperatures influence pathogen survival, replication, and spread. Diarrhea persists as a leading cause of global child morbidity and mortality and was responsible for 9 percent of deaths in children globally in 2021, 60% of which were attributable to contaminated drinking water and poor hygiene.^48^ Many studies demonstrate a pattern of association between higher ambient temperatures and clinic visits, emergency room visits, and hospital admissions for diarrhea, with children at highest risk.^49^, ^50^, ^51^, ^52^, ^53^ A case-crossover study in Brazil from 2000 to 2015 investigated variation in hospitalizations for GI infection for every 5-degree Celsius increase in mean daily temperature. With hotter mean temperatures, infants experienced the greatest increased risk of hospitalization due to GI infection, with the largest effects seen for waterborne and foodborne infections.^54^

#### Foodborne and waterborne disease

Increased temperatures and heavy precipitation driven by climate change influence the incidence of certain food-borne pathogens, including *Campylobacter*, *Escherichia coli*, and *Salmonella*.^55^, ^56^, ^57^, ^58^ In a 12-year time series study in Israel, infants and children 0–2 years of age demonstrated a linear 5.1% (95% confidence interval [CI]: 2.1%–8.1%) increase in *Campylobacter* infections for every 1-degree Celsius increase in ambient temperature,^59^ an association hypothesized to be due to influences of higher ambient temperatures on human eating patterns and poultry contamination.^58^^,^^59^ Higher ambient temperatures have also been associated with higher rates of *E coli* and *Salmonella*, which can be partly attributed to improved survival and proliferation conditions of these pathogens in warmer temperatures.^58^^,^^60^^,^^61^

Furthermore, flooding and elevated temperatures can affect the incidence of certain waterborne diseases, such as *Vibrio* spp., by enhancing conditions for growth. The suitability of coastal waters for *Vibrio cholerae* has increased by 9.9% since the early 1980s due to sea surface temperature changes driven by climate change.^62^ *Vibrio vulnificus*, a cause of severe wound and GI infections in humans, is also climate-sensitive. A study of *V vulnificus* wound infections found 8 times greater incidence in 2018 than in 1988 as well as a northern shift in cases.^63^ The authors project that case numbers may double by mid-century in anticipated climate scenarios.^63^ While the study focuses on wound infections, similar increased risk may manifest for *V vulnificus* GI disease. Additionally, flooding can contaminate groundwater, mobilize pathogens from human or animal feces, resuspend pathogens present in sediment, and overwhelm capabilities of local water treatment infrastructure.^64^^,^^65^ Children in areas impacted by flooding, heavy rainfall, or densely populated postdisaster settlements are at risk for enteric infections spread via the fecal-oral route.^66^^,^^67^ Local rainfall and population density have been associated with risk of childhood diarrhea caused by infection with *Cryptosporidium*, a top cause of parasitic diarrhea in children globally.^68^

In contrast to the bacterial pathogens discussed above, the incidence of norovirus, a significant cause of gastroenteritis in the cooler months, demonstrates an inverse relationship with ambient temperature.^69^^,^^70^ At the same time, climate-driven changing precipitation patterns and extreme weather events also influence pathways that could increase risk for norovirus transmission–environmental interactions that are challenging to characterize.^71^

While heat and heavy rainfall are more well-studied climate drivers of diarrheal illness, there have also been documented outbreaks of infectious diarrhea due to pathogens such as *E coli* and cholera associated with drought conditions.^72^^,^^73^ Potential mechanisms for this have been discussed by Levy et al,^65^^,^^74^ but most notably point to higher concentrations of pathogens in water, increased water sharing, and reduced hygiene. Treatment of drinking water and safe access to clean water are crucial for decreasing the incidence of child GI illness due to waterborne diseases.^75^

### Vector-Borne Disease, Biodiversity Loss, and Implications for Future Pandemics

Several studies have investigated the role of climate change in increasing the spread of vector-borne diseases with GI manifestations, such as malaria, dengue, Zika, and schistosomiasis.^76^^,^^77^ Climate-driven increases in precipitation and temperature, along with rising sea levels, ultimately contribute to a wider geographic range of vector habitability and reproduction, longer disease-transmission seasons, and changes in host and reservoir dynamics.^78^ These health impacts are heightened when significant events (eg, extreme weather events or pandemics) contribute to health-care system vulnerabilities, such as strained health-care resources and limited infection prevention capabilities.^79^

Deforestation from wildfires and human encroachment in undeveloped areas are contributing to the planet’s loss of biodiversity on a massive scale. Increased exposure to animals with human expansion increases risk for future pandemics like COVID-19, and biodiversity loss can further contribute to future pandemics through relative increases in host animal populations.^8^^,^^80^ Typically, zoonoses spread initially among animal hosts, though increased human-animal interaction as a result of increasing urbanization together with other climate drivers of infectious disease can precipitate outbreaks.^81^ Strategically allocating resources toward informed surveillance strategies can mitigate risk of the emergence and spread of these infectious diseases.^81^

### Climate Change Impacts on Food Security and Nutrition

Of particular relevance to childhood GI health, the “global syndemic” refers to the cumulative effects of 3 separate pandemics–obesity, undernutrition, and climate change. Climate change is fueling extreme weather events and ecosystem changes that are threatening human health and upending human systems across the globe—a scale of pandemic proportions. Often considered in isolation, these challenges of nutrition and climate change share common drivers at the individual and population levels.^82^ While food production contributes to climate change, through land transformation and fossil fuel pollution, climate change is also an important driver of food insecurity, with risks for child health and nutrition. Climate change is affecting agricultural productivity, the soil microbiome, biodiversity, and nutrient quality and quantity in crops—and by extension, the availability of nutritive foods for children that are crucial for their growth and development. Indirect pressures, including increased fuel costs, conflict, and political issues, further compound these challenges.^83^

### Nutrients in Crops and Child Malnutrition

Undernutrition contributes to almost half of global child mortality,^84^ and climate change risks worsening child nutrition. Children require both adequate caloric energy and appropriate amounts of macro and micronutrients to meet their growth and development needs. Malnutrition manifests with poor long-term physical and psychosocial outcomes for children. Malnutrition can contribute to short stature and poor bone development in children and adolescents. These childhood impacts can have intergenerational effects as they are linked to adverse obstetric outcomes from cephalopelvic disproportion and risk for obstruction during labor.^85^ A study of children in sub-Saharan Africa found a strong negative relationship between child weight measures and average annual temperature, with weight decreasing steeply with temperatures above 25 °C.^86^ This effect was particularly pronounced in children living in rural areas where crop yield was negatively affected and was more transient for children living in urban areas where it was thought to be more reflective of the association between higher temperatures and diarrhea.^86^

Climate change can also affect the growth and nutrient composition of common foods. C3 crops (eg, wheat, rice, soybeans, oats) are important to global food security and nutrition. While these crops may benefit from additional CO_2_ in the environment, many are vulnerable in extreme heat and drought scenarios. Increased levels of CO_2_ also have been associated with reduced micronutrient (eg, iron and zinc) content in these crops, as well as reduced protein composition in nonlegume C3 crops.^87^

### Climate Change Implications for Global Food Security

While the Green Revolution of the 20th century dramatically improved global food productivity and the efficiency of crop yields, continued global population growth projected through the last quarter of this century counterbalances—and climate change risks undermining—these technological advances.^88^ Climate-driven extreme weather, including storms, flooding, fires, and drought, threaten agriculture yields and disrupt food transport and storage. As the demand for food rises with a growing population, human-driven pressures on planetary boundaries threaten supply.^89^ Food scarcity due to climate change and disasters can also drive-up prices of staple food items and lead to food desserts in higher income countries.^8^^,^^89^ Simultaneously, overconsumption of nutritionally poor food, linked with food insecurity, can result in obesity.^8^

Results from a study analyzing the impacts of climate change on food insecurity in 83 countries suggest that for every 1-degree Celsius increase in temperature, moderate to severe global food insecurity increased by 1.58% (95% CI: 1.48–1.68) in 2014 and 2.14% (95% CI: 2.08–2.20) in 2019, controlling for Human Development Index and drought, suggesting that climate change’s multifactorial impact on food insecurity is increasing.^90^ Climate change is projected to contribute to a 3.2% per-person reduction in food availability and 4.0% reduction in fruit and vegetable consumption by 2050, altogether associated with over half of a million deaths worldwide, most occurring in South and East Asia.^91^ Another modeling study estimates that climate change will have the greatest impact on rates of severe stunting as an indicator of malnutrition, with an estimated increase by 23% in central sub-Saharan African and 62% in South Asia by 2050.^92^ These and other studies forecast the vulnerability of children to the impacts of climate change on agricultural productivity.

These projections can guide public health initiatives and tools to secure access to food and nutrition programming, dietary education, and more plant-based meal options for children at school. For example, novel tools that assess the effect of climate change on agricultural production and livelihoods may encourage climate resilience in food production. The agriculture livelihood vulnerability index was developed in the context of a study in coastal Bangladesh.^93^ It measures biophysical, agroecological, and socioeconomic factors associated with agricultural vulnerability to climate change. While it includes factors such as temperature change, precipitation variability, and disaster hazards, it also considers number of schools and enrollment rates, percent of growth stunted children, and infant mortality as weighted indicators of vulnerability.^93^ Partnership between public health and GI professionals can help inform these tools and optimize climate resilience in nutrition.

Furthermore, child health outcomes related to these climate-related pressures on food security underscore the interplay between genetic and environmental factors. As an example, MASLD prevalence in the United States is highest among Hispanic individuals, which is partially attributed to a single nucleotide polymorphism in the *PNPLA3* gene, in addition to metabolic and socioeconomic factors.^94^^,^^95^ In a study of 153 Hispanic children ages 8–18 years, children with this variant of the *PNPLA3* gene were the only genotype group to have a significantly higher hepatic fat content when their diet included high carbohydrate and total sugar intake.^96^ In a prospective cohort study of 136 Latinx children, food insecurity at age 4 years was associated with a 3.7-times higher risk of developing MASLD later in childhood.^97^ Families facing food insecurity often have limited access to more nutritionally rich foods and may rely on dietary options with high sugar content. GI professionals should consider both genetic and environmental risk factors of their patients in MASLD assessment and management.

### Planetary Health Diets

As climate change threatens food security and the nutrition status of children, changes in global diets are a powerful lever to address climate change. Globally, continued attention to maternal and child nutrition, along with access to sufficient quantity and quality of nutritious foods, is critical. At the same time, in wealthier countries, dietary shifts toward more plant-forward, “planetary diets” have the potential to both improve human health and reduce carbon emissions. Meat and dairy production require more natural resources—land and water—and produce more emissions per calorie than plant-based alternatives. Cattle grazing is the leading global cause of forest land clearing for pasture. Cattle also produce methane, a greenhouse gas (GHG) that breaks down in the atmosphere more quickly than CO_2_ but is many times more potent in its global warming potential in the near term and over a 100-year timescale. Nonseasonal fruits and vegetables require measures such as greenhouse storage or air transport for cultivation, also producing more emissions than the equivalent consumption of seasonal fruits and vegetables.^82^ A large meta-analysis of global food systems estimates the many environmental implications of the current food supply system, such as GHG emissions, land use, and water use.^98^ In terms of land use, current diets require about 4.13 billion hectares of land, of which 2.89 billion hectares are used for pasture and 1.24 billion hectares are used for cropland (43% of which is used for animal feed).^98^ In contrast, global transition to fully plant-based diets would reduce total agricultural land use by 75%.^99^ While these statistics are illustrative, diets that are both vegan and dependent upon seasonal crops are not practical for much of the globe. For areas of the world that consume significant amounts of meat, steps toward increased intake of plant-based nutrients and reduced meat consumption are still meaningful.

The EAT-Lancet Commission Report highlights specific dietary recommendations with the potential to provide both health and environmental benefits.^100^ The report establishes target goals focused on affordable, healthy food options, largely plant-based and with a reduction of red meat and sugars by almost 50%. Without intervention, future generations of children may have access to a progressively degraded version of this planet’s ecosystems, including conditions that challenge the ability of food supply to keep up with demand. Inequities in food supply and nutrition demand global, multisector collaboration and attention to climate resilience among agriculture, maternal and child health, food aid, and nutrition programs.^101^ Plant-forward diets in these settings can be especially beneficial to individual child health and global reduction of GHGs.^83^

### Emerging and Increasing Threats to Gastrointestinal Health

#### Harmful algae blooms

Nutrient pollution (ie, nitrogen and phosphorus) from agricultural and industrial runoff, warmer water temperatures, and extreme weather events (eg, hurricanes, floods, droughts), particularly involving coastal and freshwater regions, contribute to overgrowth of certain microscopic algal or cyanobacterial species, precipitating Harmful Algal Blooms (HABs).^102^^,^^103^ Cyanobacteria are the most common cause of HABs in freshwater systems, and many species of cyanobacteria produce cyanotoxins.^103^ In a global review of cyanobacterial illness reported in animals and humans, gastroenteritis was the most common reported health outcome from cyanobacteria exposure through ingestion and recreational water contact, and hepatotoxicosis was one of the top 2 reported syndromes from ingestion or inhalation of cyanobacteria.^104^

Between 2016 and 2018, HAB-associated illness was reported in nearly 400 people in the United States, with approximately 40% of cases in children.^105^ In case studies of HAB-related illness, morbidity and mortality rates were higher in children compared to adults, a finding attributed to children’s physiology and development, as well as behavioral factors (eg, swimming, swallowing water, ignoring posted warnings).^106^ Participation in recreational water activities typically increases with warmer temperatures, and young children who are already more vulnerable to effects of both HABs and infectious waterborne illness may face increased exposure to these hazards from an early age in a changing climate.^107^

Among cyanotoxins, microcystins and nodularins are established hepatotoxins, and microcystins can also contribute to increased intestinal cell apoptosis.^104^^,^^108^^,^^109^ Microcystins are thought to be hepatotoxic by disrupting lipid and glucose metabolism in the liver, inducing hepatocyte apoptosis, and activating inflammatory signaling pathways.^110^ There is also emerging evidence that microcystins may exacerbate pre-existing GI diseases, such as MASLD and IBD.^108^ In a mouse model of MASLD, chronic low-dose exposure to Microcystin-LR [Leucine (L) and Arginine (R)] resulted in increased hepatic injury.^111^ In a separate study performed on MASLD mice, a change in the gut microbiome was observed following exposure to microcystin, which was further associated with intestinal epithelium erosion, crypt abscesses, and granulated cells compared to controls, suggesting a possible environmental mechanism for exacerbation of MASLD-associated intestinal comorbidities.^112^ The compound effects of heat stress and microcystins on the gut microbiome and intestinal permeability may also increase the virulence of certain pathogens by allowing for endotoxin absorption.^113^ In mice, early life exposure to microcystins has also been associated with gut dysbiosis and expansion of antibiotic resistance genes in the gut.^114^ This could have implications for antimicrobial treatment options for bacterial infections, though this has not been well studied in humans, specifically in relation to microcystin exposure.

Pediatric GI health providers can expect to see more cases of illness due to HABs in the future and thus should be attuned to the signs and symptoms of acute cyanobacterial poisoning and educate patients about the risks of HABs.

### Climate Change and the Gut Microbiome

The gut microbiome aids in digestion and metabolism, plays an important role in preserving the integrity of the intestinal epithelial layer, and helps regulate aspects of the immune and nervous systems.^115^ The microbiome undergoes most of its evolution and diversification early in life, resembling that of an adult by about 3 years of age.^116^ Emerging literature suggests that children’s exposure to the natural environment may support a healthy gut microbiome.^117^ Multiple factors, such as diet, medications, infections, and chronic stress, can contribute to imbalances in the growth of beneficial and nonbeneficial microbes, termed dysbiosis.^118^, ^119^, ^120^ Dysbiosis has been associated with GI disorders including IBD, irritable bowel syndrome (IBS), and liver disease, in addition to obesity, diabetes, cardiovascular disease, and certain neurologic disorders.^121^

The pathways through which climate change can influence the gut microbiome are multifactorial and overlapping—if indirect—and include climate-driven impacts on extreme weather, food availability, biodiversity, and the prevalence of enteric pathogens. A cohort study assessing the post-Hurricane Maria impact in Puerto Rico in utero found that the abundance of a specific genus of generally beneficial gut bacteria, *Veillonella spp.*, was significantly decreased in infants whose mothers experienced food insecurity in the aftermath of Hurricane Maria compared to those who did not.^122^ Similarly, preschool-aged Malaysian children amid El Niño in 2016 experienced a decrease in beneficial gut microbiota, *Faecalibacterium* and *Veillonella*, and a 2-fold increase in fecal secretory IgA and *Bacteroides*.^123^ The decrease in beneficial microbes was not observed in children taking a probiotic during this time, highlighting a possible intervention to protect against dysbiosis in the wake of extreme weather events.^123^ Litchman reviews the nuances of climate change’s impacts on the microbiome more detail.^124^ Amid the many factors that influence the gut microbiome, including antibiotic use, the clinical significance of climate-driven effects are challenging to isolate and not yet well-characterized. Future research may better delineate the ways in which climate change-related environmental exposures and their associated health implications (eg, adverse birth outcomes, gut microbiome changes, and epigenetic changes) contribute to GI morbidity in the pediatric population, as well as strategies to prevent exposures and harm.

### The Gut-Brain Axis and Functional Gastrointestinal Disorders in the Climate Crisis

#### Climate change and mental health

A growing body of literature details the effects of climate change on mental health. The effects of climate change on mental health manifest differently in children than in adults.^125^ Acute events, such as extreme weather, floods, and wildfires, can increase risk for development of attachment problems, post–traumatic stress disorder, depression, and anxiety in children and adolescents.^125^^,^^126^ While the mental health impacts of climate disasters may be most apparent, downstream and indirect mental health implications of climate change for youth can stem from disruptions in school, recreation, and social activities, as well as erosion of social cohesion and resources.^125^^,^^127^ A multistudy analysis found that more than half of youth are very or extremely worried about climate change.^128^

Emerging literature supports that climate-related mental health concerns can manifest as functional gastrointestinal disorders (FGIDs) or gut-brain axis disorders.^129^ Psychological stress can also worsen colitis and trigger flares of IBD in children and adults.^130^^,^^131^ Furthermore, climate change-related mental health effects can contribute to increased substance use, which typically peaks in late adolescence and can contribute to alcohol-related GI disease and viral hepatitis.^132^, ^133^, ^134^

Health indicators related to psychological stressors amplified by climate disasters and extreme weather can be traced to the prenatal period. One study found that in utero exposure to maternal stress due to severe floods predicted greater increase in a child’s total adiposity between 2.5 and 4 years of age.^135^ While adiposity itself is not a GI disease, adiposity is a risk factor for a range of adverse GI outcomes.

### Mechanisms for Environmental Influence on Functional Gastrointestinal Disorders

Children with FGIDs experience symptoms from abnormal function of the GI tract without a clear organic etiology.^136^ Mental health and FGIDs are closely linked, and patients with FGIDs have an increased prevalence of psychiatric disorders compared to healthy controls.^137^ FGIDs are thought to occur due to an interaction of biologic, psychologic, social, and environmental factors.^136^ FGIDs can lead to significant functional impairment and impact quality of life^138^; however, treatment of mental health challenges can help alleviate somatic symptoms.^136^ IBS is one of the most common FGIDs. An association between climate change-related mental health impacts and somatic symptoms has been documented. In a study evaluating youth somatic complaints during a hurricane-related evacuation, predisaster evacuation stressors were indirectly associated with increased somatic complaints, such as headaches, nausea, vomiting, and stomachaches, through youth psychological distress.^139^

Researchers have hypothesized that the associations between climate change, psychosocial stressors, and FGIDs may stem from (1) exposure to air pollution and (2) climate-driven increases in enteric infections.^140^ As discussed above, air pollution can increase systemic inflammation, contribute to oxidative damage of colonic mucosa, and alter the gut microbiome—cascading effects on the GI tract that may also contribute to FGIDs.^140^ In a cohort study of Taiwanese children stratified by daily average air pollutant exposure, the incidence of IBS significantly increased with higher levels of exposure to 4 pollutants, most notably methane.^141^ In a study in Montreal and Edmonton, Canada, emergency department visits for nonspecific abdominal pain were most common in 15–24-year-olds, predominantly women, and significantly more likely with elevated ambient levels of specific pollutants (carbon monoxide and NO_2_ in both locations, and sulfur dioxide and fine particulate matter [2.5 microns or less in diameter] in Montreal).^142^

As climate change drives extreme weather events and disrupts infrastructure, increased incidence of enteric infections in the setting of heavy rainfall or lack of access to clean water may result in increased incidence of postinfectious IBS (post–infection irritable bowel syndrome). In adult Chinese patients without history of FGID who were followed up to 2 years after infection with bacillary dysentery, there was a significantly higher incidence of IBS among those infected compared to controls.^143^ In the same study, duration of infection (>2 weeks vs ≤1 week) was associated with higher likelihood of post–infection irritable bowel syndrome.^143^ Integral to the enteric nervous system, rectal mucosal enteroendocrine cells may increase after gastroenteritis, resulting in changes in mucosal barrier function that could partially explain postinfectious GI symptoms.^144^

The links between environmental exposures and FGID remain largely indirect, nonspecific, and similar to associations observed with other systemic causes of increased stress. Ultimately, more research is needed to better elucidate the complex relationship between functional GI diseases, mental health, and climate change. In practice, identifying patients’ climate-related mental health vulnerabilities through environmental history and screening for displacement and disaster exposure, as well as considering mental health treatment for patients when necessary, should be considered in comprehensive care of childhood FGID and other GI disorders.

### Climate-Driven Migration and Disasters

Climate-driven disasters also influence GI health by disrupting access to health care and via downstream effects on forced displacement and migration.^145^ Disasters like hurricanes and cyclones, wildfires, and floods are increasing due to climate change, and many affect hospitals, clinics, and health-care delivery infrastructure. Disasters can also cause or contribute to supply shortages of common and specialized formulas and foods for infants and children with special dietary needs. When disasters impede access to essential health-care or critical supplies, children who depend on specialty care or specialized medical equipment (eg, special formulas, gastric tubes, or feeding pumps), face increased risk of harm. Clinicians can review disaster preparedness plans with their patients—especially those who depend on special formula or feeding equipment—at routine visits to ensure appropriate preparedness plans are in place.

Climate-driven disasters are also contributing to the global movement of people. From 2016 to 2021, UNICEF reports that 43.1 million children, approximately 20,000 children daily on average, were displaced due to weather-related disasters, 95% of which consisted of floods and storms.^146^ Not only acute events but also longer-term climate stressors contribute to the movement of people globally. Families may move in response to long-term environmental changes that lead to food insecurity, lack of access to drinking water or sustainable agriculture, rising temperatures, or inhabitability of lands due to rising sea levels.^147^ In a meta-analysis of 30 studies investigating the effects of environmental factors on migration, the greatest effects were due to temperature, natural disasters, and variability and anomalies in rainfall patterns.^148^ The decision for families to migrate can be complex, with resource limitations due to effects of climate change interplaying with economic, social, and political factors. The ongoing Syrian conflict, thought to be catalyzed by a severe drought that prompted increased migration, is an often-cited example of this complexity.^149^

Both migration and displacement due to climate-driven severe weather events or long-term environmental changes can affect children’s gut health through several mechanisms. Migration is often accompanied by health challenges during travel or upon resettlement. Most centrally, malnutrition can be both a cause and result of migration in the context of food insecurity.^150^ Additionally, displaced children are at risk for infectious diarrhea, especially in low-resource, densely populated urban centers or refugee camps.^151^^,^^152^ Internally displaced children may face limited access to essential health-care needs, including treatment for acute and chronic GI illnesses.^153^ Children faced with displacement or threat of displacement from their home are at risk for developing mental health conditions, such as depression, anxiety, and post–traumatic stress disorder, which are known to interplay with FGIDs.^147^ When caring for patients from migrant or refugee families, GI professionals should consider unique risk factors from their place of origin, potential exposures during migration, and risks upon resettlement.

## Conclusion

The children of current and future generations are increasingly vulnerable to the impacts of climate change. It is therefore increasingly important for the GI community to recognize the short-term and long-term effects on children, as well as the environmental risk factors of their patients. Action can be taken at multiple levels of prevention (Figure 2), and there are practical ways for GI providers to engage in these efforts to support child health (Figure 3).

**Figure 2:**
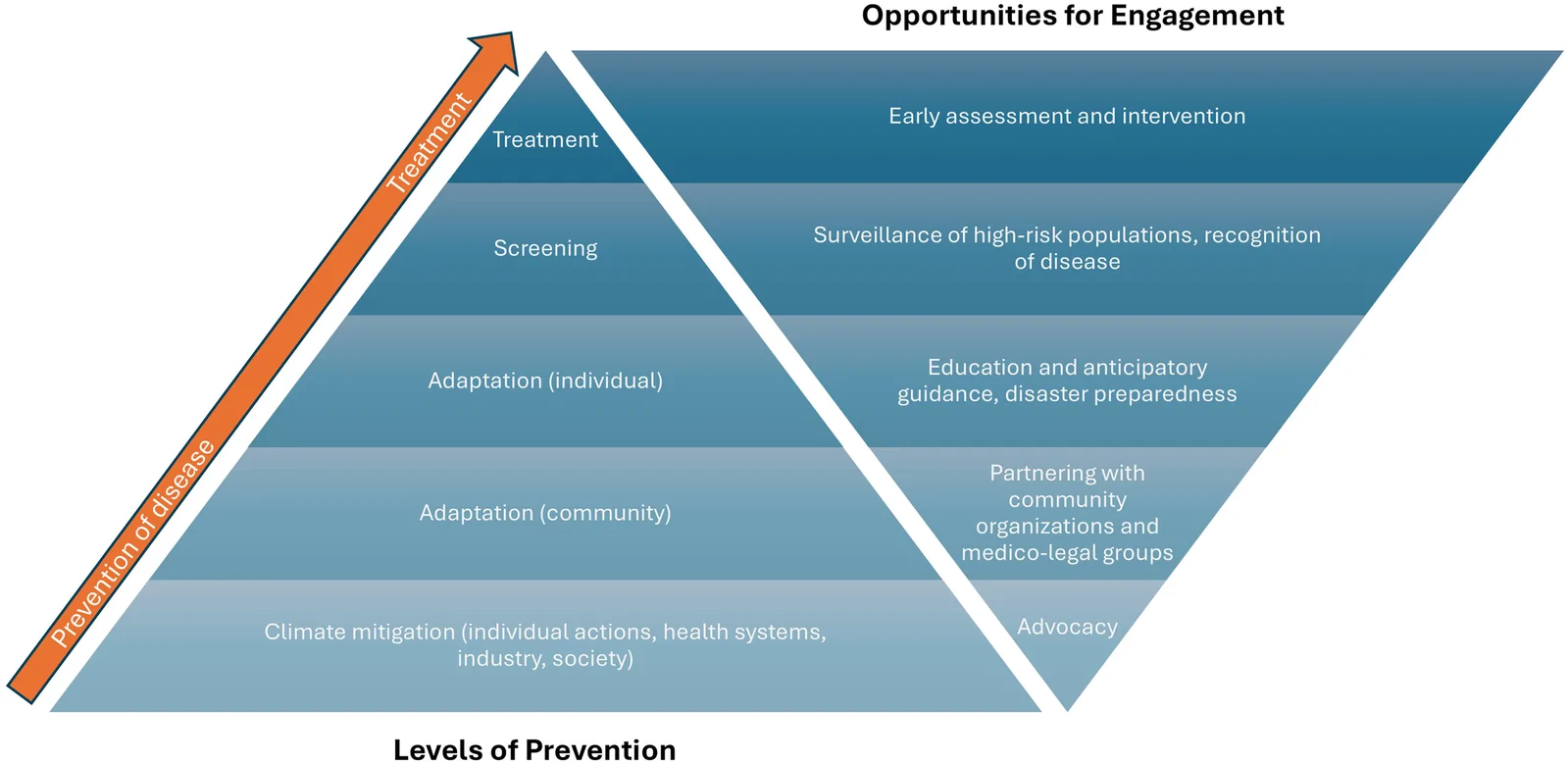
Levels of prevention and opportunities for gastrointestinal provider engagement.

**Figure 3:**
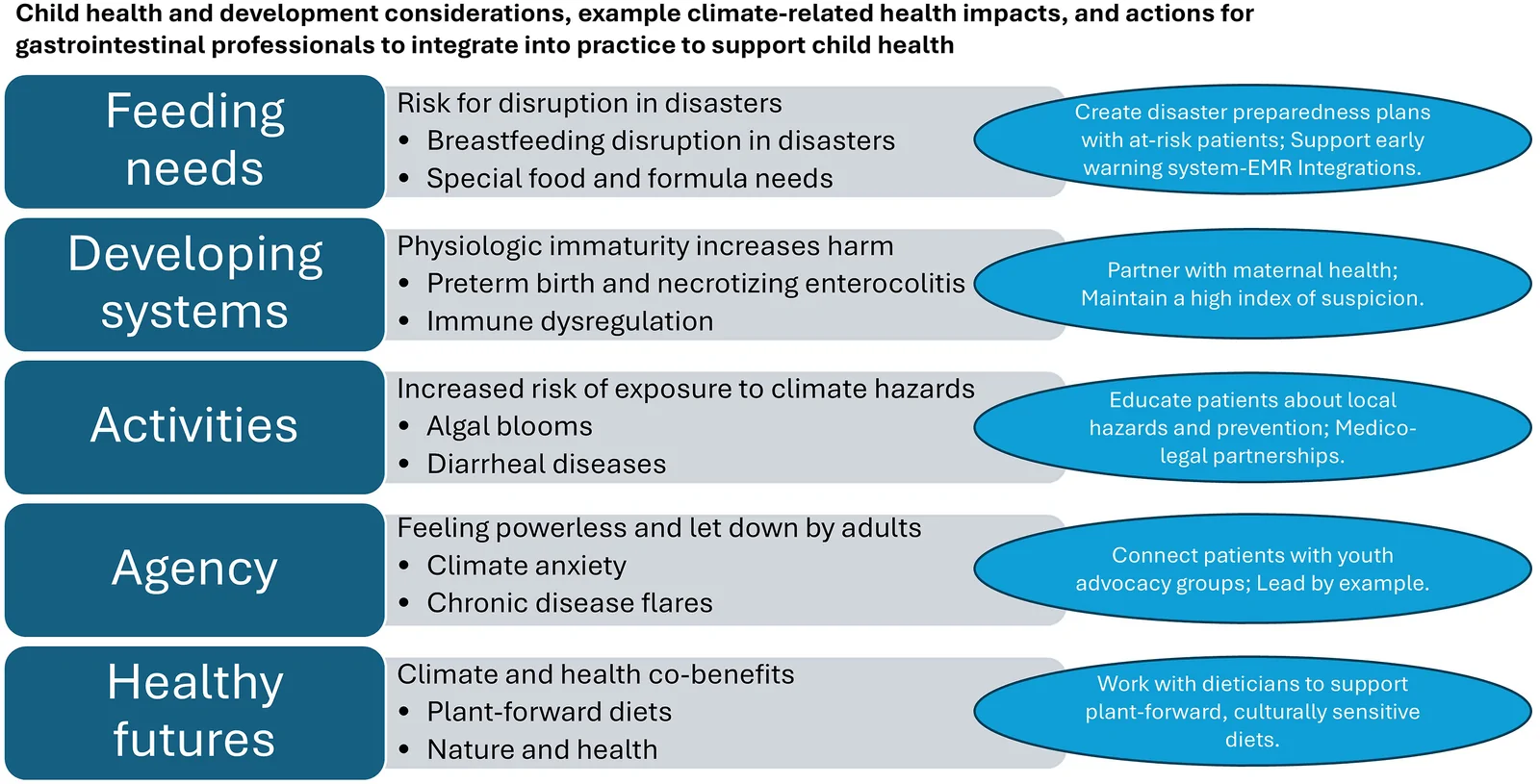
Child health and developmental considerations, example climate-related health impacts, and actions for gastrointestinal professionals to integrate into practice.

Many national and international groups have formed within the GI community to work toward reducing environmental impacts associated with health-care delivery.^8^^,^^154^ Tracking, reporting, and sharing data regarding environmental impacts on child health and successful interventions across and within regions will be essential for climate resilience.^155^^,^^156^ Surveillance systems can detect and inform communities of climate threats.

Health systems need to be aware of and prepared to respond to climate change-related health effects, including promotion of care for vulnerable communities, such as immigrants, refugees, and displaced children.^157^^,^^158^ Pediatric clinicians can incorporate climate considerations into health visits, practicing climate-informed care, and screen families for social determinants of health that may reflect impacts of as well as vulnerability to effects of climate change.^159^ Furthermore, pediatric gastroenterologists can play a crucial role in listening to and advocating for children who are particularly vulnerable due to chronic conditions and special health-care needs. Ensuring availability of community resources and access to special dietary and nutrition needs (including school food and nutrition programs), as well as implementing disaster plans with access to public health alerts, can improve the resilience and adaptive capacity of children in the face of climate change.^1^

This review is limited by the paucity of evidence on the impacts of climate change on GI health as well as studies focusing on the pediatric population. Many studies included referred to the challenges of analyzing climate-associated GI concerns in isolation, as outcomes are often multifactorial.

While we see and experience the impacts of climate change unfold, further research is needed to better understand impacts on the GI and overall health of children. While this review focuses on patient care intersections, pediatric GI professionals have an opportunity as a community to engage in the needed health-care sector transformation toward sustainable care delivery, bolster the resilience of health-care infrastructure, and advocate for policies to protect pediatric patients. Now is a critical time for action toward a more sustainable, safe, and adaptable planet for our children and future generations.
